# Accurate Evaluation of Vertical Tidal Displacement Determined by GPS Kinematic Precise Point Positioning: A Case Study of Hong Kong

**DOI:** 10.3390/s19112559

**Published:** 2019-06-05

**Authors:** Guoguang Wei, Qijie Wang, Wei Peng

**Affiliations:** School of Geosciences and Info-physics, Central South University, Changsha 410083, China; 175011007@csu.edu.cn (G.W.); pengweicsu@csu.edu.cn (W.P.)

**Keywords:** KPPP, tidal displacement, accuracy

## Abstract

Global Positioning System (GPS) kinematic precise point positioning (KPPP) is an effective approach for estimating the Earth’s tidal deformation. The accuracy of KPPP is usually evaluated by comparing results with tidal models. However, because of the uncertainties of the tidal models, the accuracy of KPPP-estimated tidal displacement is difficult to accurately determine. In this paper, systematic vector differences between GPS estimates and tidal models were estimated by least squares methods in complex domain to analyze the uncertainties of tidal models and determine the accuracy of KPPP-estimated tidal displacements. Through the use of GPS data for 12 GPS reference stations in Hong Kong from 2008 to 2017, vertical ocean tide loading displacements (after removing the body tide effect) for eight semidiurnal and diurnal tidal constituents were obtained by GPS KPPP. By an in-depth analysis of the systematic and residual differences between the GPS estimates and nine tidal models, we demonstrate that the uncertainty of the tidal displacement determined by GPS KPPP for the M2, N2, O1, and Q1 tidal constituents is 0.2 mm, and for the S2 constituent it is 0.5 mm, while the accuracy of the GPS-estimated K1, P1, and K2 tidal constituents is weak because these three tidal constituents are affected by significant common-mode errors. These results suggest that GPS KPPP can be used to precisely constrain the Earth’s vertical tidal displacement in the M2, N2, O1, and Q1 tidal frequencies.

## 1. Introduction

The tidal force of the Sun and Moon can cause deformation of the solid Earth, which is named “body tide”, and it also causes periodic rise and fall of the ocean mass, which is called “ocean tide”. The response of the solid Earth to this mass distribution is known as “ocean tide loading” (OTL). Modern high-precision geodetic technology can determine large-scale deformation at the sub-millimeter level, but the effect of tidal displacement must be considered [1,2,3,4]. Body tide can now be modeled with an accuracy of usually less than 1% [5]. OTL displacement can be obtained by convolution integral of an ocean tide model and the Green’s function calculated from an Earth model [6], which we call an OTL model. Penna et al. [7] reported that the height error of M2 OTL displacement can reach around 20% (~8 mm), depending on the ocean tide model adopted and the handling strategy for the grid cells in coastal areas. The change of the vertical OTL displacement in some areas when using various Earth models can reach 2–3 mm [8]. Therefore, OTL is still the main uncertain factor in tidal displacement analysis.

Since 2000, the Global Positioning System (GPS) has been widely used to estimate OTL displacement, including eight semidiurnal and diurnal tidal constituents. The methods for estimating OTL displacement can be divided into static and kinematic methods. Adding OTL displacements (amplitude and phase) to the static (usually one-day) GPS solution model and estimating them together with other parameters such as coordinates is called a static method. Schenewerk et al. [9] first determined the vertical OTL displacements by a static method, and reported that the amplitude differences of the M2 height component between the GPS estimates and the OTL model were less than 5 mm at 90% of the GPS reference stations. Over the next 10 years, many scholars [10,11,12,13] used static methods to estimate OTL displacements in different areas, for which the M2 vector difference between the GPS estimates and the OTL model can reach the sub-millimeter level. Yuan et al. [14] estimated global OTL displacements, which indicated that the vertical root-mean-square (RMS) residuals of M2 were 0.6–1.2 mm between the GPS estimates and the different OTL models.

Estimating OTL displacement from a high-sampling GPS coordinate series is called the kinematic method. Khan and Tscherning [15], Khan and Scherneck [16], Yun et al. [17], Melachroinos et al. [18], and Vergnolle et al. [19] used a GPS (1–4 h) relative coordinate series to estimate relative OTL displacements in different regions, for which the M2 vector difference between the GPS estimates and OTL models was usually 1–5 mm. King et al. [20] were the first to estimate absolute OTL displacements in Antarctica using a GPS kinematic precise point positioning (KPPP) coordinate series every 300 s. Since then, Ito et al. [21], Ito and Simons [22], Bos et al. [8], and Tu et al. [23] have used KPPP to estimate the tidal deformation in different areas. These authors reported that the vertical M2 vector difference between the GPS estimates and OTL models can reach the sub-millimeter level. Zhao et al. [24] used an 18.6-year KPPP series to estimate OTL displacements at Shanghai Astronomical Observatory (SHAO) station in China, and reported an M2 RMS agreement of around 0.6 mm in the vertical component compared with OTL models.

In previous studies, the accuracy of GPS-estimated OTL displacement has been evaluated by comparing the results with OTL models. However, this is not accurate enough, because the difference between GPS estimates and OTL models also includes the uncertainties of the OTL models, the unmodeled body tide, the numerical calculation error, and so on. Penna et al. [25] added synthetic signals with an amplitude of 0–6 mm and a period of 13.96 h to a 4-year KPPP coordinate series and demonstrated that the added signals could be determined with an uncertainty of 0.2 mm. However, whether the accuracy of the KPPP estimates of the eight semidiurnal and diurnal tidal constituents can reach 0.2 mm needs to be further validated.

**Figure 1:**
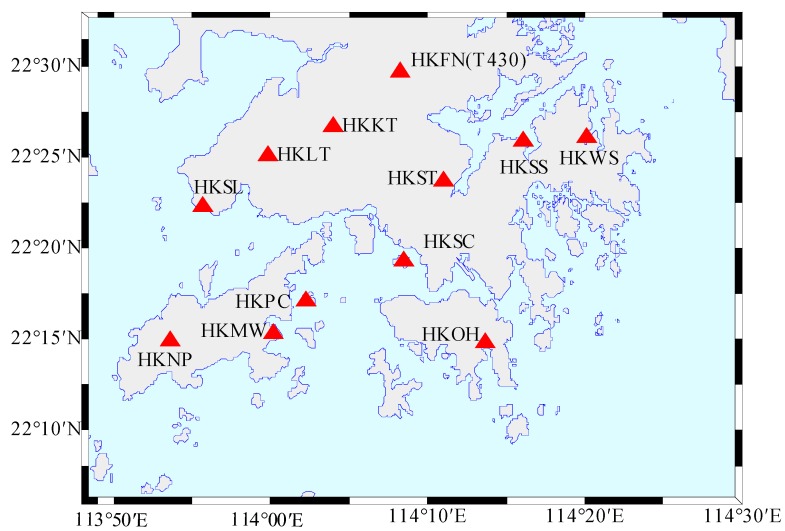
Distribution of the GPS reference stations in the Hong Kong area.

In a small region, the unmodeled body tide and the uncertainties of the OTL model have almost the same effect on all the GPS reference stations, which can be modeled as a systematic component for all the stations. In this study, the systematic and residual components at all the stations were obtained by least squares methods, and used to evaluate the accuracy of the eight KPPP-estimated semidiurnal and diurnal tidal displacements. The Hong Kong area has many uniformly distributed GPS reference stations (Figure 1), so it was chosen as the research area in this study. Through the GPS-observed data from 2008 to 2017, an in-depth study was conducted that how precisely GPS KPPP can provide constraints for the eight semidiurnal and diurnal tidal constituents.

## 2. Data Processing and Analysis Method

### 2.1. Method of KPPP-estimated OTL Displacement

The influence of OTL in the horizontal direction is about one-third of that in the vertical direction [8]. Therefore, in this paper, we mainly analyzed the OTL displacement in the vertical direction. According to the International Earth Rotation and Reference Systems Service (IERS) Convention 2010 [26], the vertical OTL displacement of a station can be represented by four semidiurnal (M2, S2, N2 and K2) and four diurnal (K1, O1, P1 and Q1) tidal constituents, depending on the frequencies:

(1) $$\Delta c=\sum_{j=1}^{8}f_{j}A_{cj}\cos(\omega_{j}t+\chi_{j}(t_{0})+u_{j}-\phi_{cj})$$

where $A_{cj}$ and $\phi_{cj}$ respectively represent the amplitude and phase of the *j*-th tidal constituent at the station; $f_{j}$ and $u_{j}$ denote the nodal modulation corrections; $\omega_{j}$ is the angular frequency; and $\chi_{j}\left(t_{0}\right)$ is the astronomical argument at reference time $t_{0}$. Table 1 lists the periods of the eight semidiurnal and diurnal tidal constituents. For a given location, except that the amplitude and phase of the *j*-th tidal constituent need to determined, the remaining parameters can be obtained according to the expression of these arguments in the IERS Convention 2010 [26]. 

GPS kinematic precise point positioning (KPPP) is an absolute positioning method, which can directly determine the position of a receiver based on satellite constellations [27]. Therefore, an absolute displacement series in vertical direction with a high sampling rate can be obtained by the GPS KPPP solution. OTL displacements can usually be effectively estimated by using a 4-years of GPS observation [25], but simultaneous nodal correction is also needed. The OTL amplitude and phase of the semidiurnal and diurnal tidal constituents can be estimated from the KPPP coordinate series by using the classical harmonic analysis method [28].

### 2.2. GPS Data Acquisition and Processing

Hong Kong is located on the north coast of the South China Sea, and is mainly composed of the Kowloon Peninsula and many islands. The Hong Kong coastline is complex and is affected by OTL. GPS observation data for 2008–2017 provided by the Surveying and Mapping Office of the Hong Kong Lands Department (https://www.geodetic.gov.hk/smo/gsi/programs/tc/index.htm) were used, in which the HKFN station was replaced by the T430 station after 2014. The distribution of the GPS reference stations in the Hong Kong area are shown in Figure 1.

By using 24-h GPS observation data in RINEX format, the kinematic PPP solution for every 600 s was carried out with Bernese GNSS software version 5.2 [29] (http://www.bernese.unibe.ch/docs/DOCU52.pdf), in which the elevation cutoff angle was set to 3°. The main parameter files, i.e., satellite orbit, 30 s satellite clock, Earth rotation, ionosphere parameter, and differential code bias (DCB), were provided by the European Center for Orbit Determination in Europe (COD). The tropospheric delay was modeled using the Global Mapping Function (GMF) [30]. The antenna phase center file used PCV_COD.I08. Body tide and pole tide were modeled according to the IERS Convention 2010 [26]. No corrections were applied to the ocean and atmospheric tide loading.

The estimated KPPP coordinates were in the IGS08 reference frame. The origin of IGS08 is defined as the center of mass (CM), including the solid Earth, the ocean, and the atmosphere, which is usually called the CM frame. Coordinates under IGS08 reference frame were transformed into the station coordinate system, including east, north, and vertical directions. The linear trend of the vertical series was removed. A threshold of 200 mm was set to eliminate gross errors. The % of Gross Errors are shown in Table 2. The eight semidiurnal and diurnal tidal components in the vertical direction at 12 stations were estimated using the method described in Section 2.1. The amplitudes and phases of OTL displacements for eight tidal constituents determined by GPS KPPP are listed in Table A1 and Table A2.

### 2.3. OTL Model and Tidal Signal Analysis

OTL displacement predicted by a model is usually based on the Green’s function [6], which is determined by the adopted Earth model and represents the response characteristics of the solid Earth to mass loading. The OTL displacement u for a location r is as follows:

(2) $$u(r)=\int_{\Omega }\rho G(|r-r{}^{\prime }|)Z(r{}^{\prime })d\Omega$$

where *Z* is a vector expression of the tide at $r^{\prime }$ provided by the ocean tide model; Ω represents the global ocean area; ρ is the density of sea water; and *G* is the Green’s function, characterizing the load deformation caused by the unit mass of sea water.

In this paper, eight recent ocean tide models were considered: FES2014b, GOT4.10c, TPXO.8, DTU10, EOT11a, HAMTIDE, NAO99b, and OSU12 [31,32]. The OTL displacements predicted by the FES2014b and GOT4.10c models based on the STW105 Earth model were provided by the International Mass Loading Service [33], for which the density of sea water was set to 1025 kg/m^3^. The predictions of the OTL displacements by FES2014b, TPXO.8, DTU10, EOT11a, HAMTIDE, NAO99b, and OSU12 were provided by the Ocean Tide Loading Provider [34], for which the density of sea water was set to 1030 kg/m^3^. The FES2014b and TPXO.8 tide models based on the Preliminary Reference Earth Model (PREM) were calculated with CARGA [7]. The DTU10, EOT11a, HAMTIDE, NAO99b, and OSU12 tide models based on the Gutenberg-Bullen A Earth model were calculated with OLFG/OLMPP [35]. A total of nine OTL models were obtained, and we refer to them as GOT4.10c + STW, FES2014b + STW, FES2014b + PREM, TPXO.8 + PREM, DTU10 + GB, EOT11a + GB, HAMTIDE + GB, NAO99b + GB, and OSU12 + GB, respectively.

**Figure 2:**
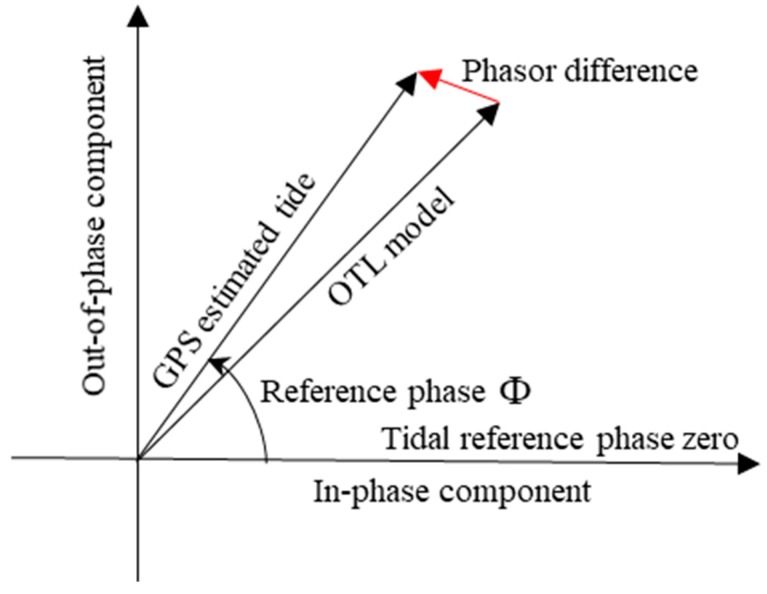
Phasor vector diagram of the relationship between the GPS estimate and OTL model.

After modeling the body tide in the GPS data processing, the GPS-estimated tidal deformation is mainly made up of the OTL displacement. A tidal constituent can be conveniently expressed in terms of the in-phase and out-of-phase amplitudes of the phasors on the complex plane [13]. The phasor vector relationship between the GPS estimate and OTL model is shown in Figure 2.

The phasor difference between the GPS estimate and OTL model for the *j*-th tidal constituent at station *k* is as follows:

(3) $$Z_{k,j}=Z_{GPS,k,j}-Z_{OTL,k,j}$$

where $Z_{GPS,k,j}$ is the tidal displacement estimated by GPS, and $Z_{OTL,k,j}$ denotes the tidal deformation predicted by the OTL model. The complex form of $Z_{k,j}$ can be written as:

(4) $$Z_{k,j}=A_{k,j}(\cos\Phi_{k,j}+i\sin\Phi_{k,j})$$

where *i* is the imaginary unit; and $A_{k,j}$ and $\Phi_{k,j}$ represent the residual amplitude and phase of the *j*-th tidal constituent, respectively.

The RMS of the phasor difference for the *j*-th tidal constituent between the GPS estimate and OTL model for N stations is calculated as follows:

(5) RMSj=(1N∑k=1N|Zk,j|2)12

In a small region, the phasor difference for the *j*-th tidal constituent between the GPS estimate and OTL model at station *k* can be divided into two kinds of components. The first kind of component is related to the specific station, i.e., the multipath effect on GPS observation. The second kind of component is applicable to all stations, i.e., the unmodeled body tide, the uncertainties of the OTL model caused by ocean tide model errors and mantle anelasticity, the GPS orbit errors, and the signal propagation delay. Therefore, the phasor difference $Z_{k,j}$ of the *j*-th tidal constituent at station *k* can be decomposed into $Z_{residual,k,j}$, which is related to the specific station, and $Z_{system,j}$, which is applicable to all stations. For the *N* stations, the phasor difference of the *j*-th tidal constituent can be expressed as follows:

(6) $$Z_{k,j}=Z_{system,j}+Z_{residual,k,j}\;\;\;(k=1,2,\cdots ,N)$$

Next, we explain how to obtain $Z_{residual,k,j}$ and $Z_{system,j}$ on the complex plane by the least-squares method. Equation (6) is written in matrix form:

(7) $$\left[\begin{array}{l} Z_{1,j}\\ \vdots \\ Z_{k,j}\\ \vdots \\ Z_{N,j} \end{array}\right]=\left[\begin{array}{l} 1\\ \vdots \\ 1\\ \vdots \\ 1 \end{array}\right]Z_{system,j}+\left[\begin{array}{l} Z_{residual,1,j}\\ \vdots \\ Z_{residual,k,j}\\ \vdots \\ Z_{residual,N,j} \end{array}\right]$$

Equation (7) can be abbreviated as:

(8) $$\underset{N\times 1}{Z_{j}}=\underset{N\times 1}{I}Z_{system,j}+\underset{N\times 1}{Z_{residual,j}}$$

where ***I*** denotes the unit matrix, and $Z_{system,j}$ is the parameter to be estimated. It is assumed that $Z_{residual,k,j}\left(k=1,\ldots ,N\right)$ obey a Gaussian distribution and are uncorrelated with each other. The expected value and variance of $Z_{residual,k,j}\left(k=1,\ldots ,N\right)$ are $E\left(\left|Z_{residual,k,j}\right|\right)=0$ and $D\left(\left|Z_{residual,k,j}\right|\right)=\sigma^{2}$, respectively. Therefore, the variance-covariance matrix of $Z_{j}$ can be written as:

(9) $$D\left(\underset{N\times 1}{Z_{j}}\right)=D\left(\underset{N\times 1}{Z_{residual,j}}\right)=\sigma^{2}\underset{N\times N}{I}$$

where D() is the variance-covariance matrix. If we let ***V*** donate the correction value of $Z_{residual,j}$ and ${\hat{Z}}_{system,j}$ represent the estimated value of $Z_{system,j}$, then formula (8) can be transformed as:

(10) $$\underset{N\times 1}{V}=\underset{N\times 1}{I}{\hat{Z}}_{system,j}-\underset{N\times 1}{Z_{j}}$$

From formula (9), we know that $Z_{k,j}\left(k=1,\ldots ,N\right)$ are equal-weight observations. Therefore, to obtain ${\hat{Z}}_{system,j}$ based on the least-squares principle, we only need to satisfy $V^{T}V=min$, in which $V^{T}$ is the transpose matrix of ***V***. In order to obtain the minimum value of $V^{T}V$, the derivative of $V^{T}V$ to ${\hat{Z}}_{system,j}$ should be 0. The detailed process is as follows:

(11) $$\frac{\partial (V^{T}V)}{\partial {\hat{Z}}_{system,j}}=2V^{T}\frac{\partial V}{\partial {\hat{Z}}_{system,j}}=2V^{T}I=0$$

According to Equations (10) and (11), the formula after eliminating ***V*** can be obtained as:

(12) $$\underset{1\times N}{I^{T}}\underset{N\times 1}{I}{\hat{Z}}_{system,j}-\underset{1\times N}{I^{T}}\underset{N\times 1}{Z_{j}}=0$$

Therefore, the least-squares estimate of complex $Z_{system,j}$ is:

(13) $${\hat{Z}}_{system,j}={\left(\underset{1\times N}{I^{T}}\underset{N\times 1}{I}\right)}^{-1}\underset{1\times N}{I^{T}}\underset{N\times 1}{Z_{j}}$$

Finally, $Z_{residual,k,j}\left(k=1,\ldots ,N\right)$ can be obtained by formula (6).

## 3. Results and Discussion

### 3.1. Comparison with the OTL Models

**Figure 3:**
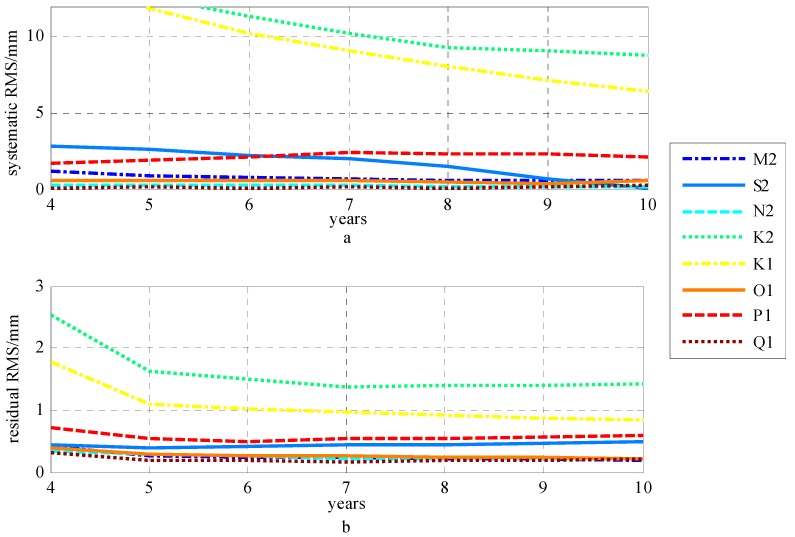
GPS-estimated tidal stability for the eight tidal constituents: (**a**) the RMS of the systematic phasor difference; (**b**) the RMS of the residual phasor difference corresponding to (**a**).

To analyze the KPPP-estimated tidal constituent stability, according to Equations (6) and (13), we calculated the residual and systematic phasor differences between the GPS estimates (using the observed data for years 4 to 10 since 2008) and the GOT4.10c + STW OTL model, for each of the eight tidal constituents at 12 stations. The corresponding RMSs of the residual and systematic phasor difference of the eight tidal constituents were obtained by formula (5), as shown in Figure 3.

**Figure 4:**
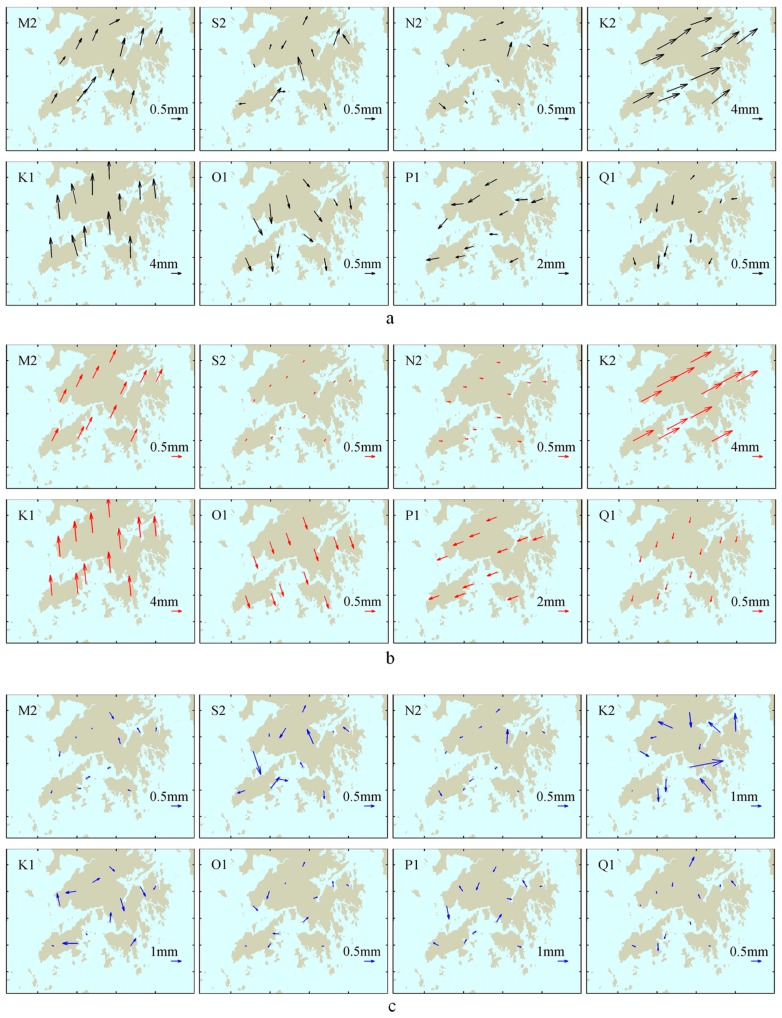
Spatial distribution of the phasor vector differences between the GPS estimates and the GOT4.10c + STW OTL model: (**a**) total phasor vector differences (black); (**b**) systematic phasor vector differences (red) estimated from (**a**); (**c**) residual phasor vector differences (blue) by (**a**,**b**).

Figure 3a,b indicate that, for the M2, N2, O1 and Q1 tidal constituents, after the fourth year, the RMS of the residual and systematic phasors for the four tidal constituents is stabilized. For the S2 tidal constituent, its residual RMS is stabilized after the fourth year; however, it is not until the ninth year that the systematic RMS of the S2 constituent begins to stabilize. Although the residual and systematic RMS of the P1 constituent is stabilized after the fourth year, the magnitude of the residual and systematic RMS for the P1 tidal constituent is significant. The residual RMS of the K1 and K2 constituents is stabilized after the fifth year, but their systematic RMS does not convergence until the tenth year. Figure 4a shows the spatial distributions of the phasor vector differences, between the GPS estimates (using the observed data for 2008–2017) and the GOT4.10c + STW OTL model, of the eight tidal constituents at 12 stations. Figure 4b shows the systematic phasor vector differences estimated from Figure 4a by formula (13) for the eight tidal constituents. The residual vector phasor differences were obtained by differencing Figure 4a,b, as shown in Figure 4c. The expression of the phasor vector difference for a given tidal constituent is given in Figure 2.

From Figure 4a, we can see that the spatial distributions of the phasor vector differences for the M2, K2, K1, O1, and P1 tidal constituents have obvious coherence. The systematic phasor differences could be well derived, as shown in Figure 4b. Figure 4a also shows that the spatial distributions of the phasor vector differences for the S2, N2, and Q1 tidal constituents had no obvious coherence; therefore, there were no distinguishing systematic differences for these three constituents, as shown in Figure 4b. Figure 4c indicates that the residual phasor differences of the eight tidal constituents had no spatial correlation, i.e., they are related to the location.

### 3.2. Accuracy Assessment for the KPPP-Estimated OTL

**Figure 5:**
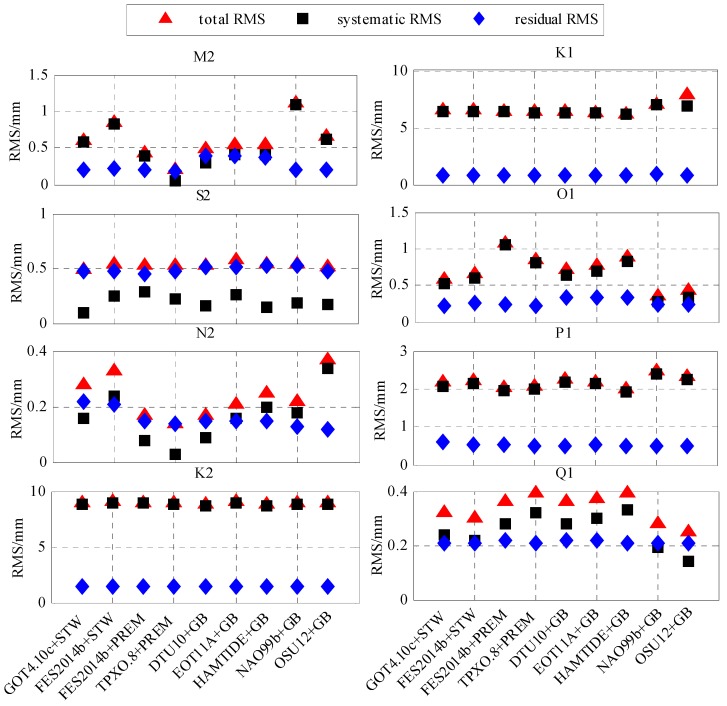
RMS values of the eight tidal constituents between the GPS estimates and the nine OTL models.

To validate the accuracy of the GPS KPPP estimates of the eight tidal constituents, according to Equations (5), (6) and (13), we calculated the RMS of the total phasor differences, the systematic phasor differences and the corresponding residual phasor differences between the GPS estimates (using the observed data for 2008–2017) and the nine OTL models, for each of the eight tidal constituents at 12 stations. The RMS values of the eight tidal constituents are shown in Figure 5.

Figure 5 shows that the systematic RMS of the N2 and Q1 tidal constituents between the GPS estimates and the different OTL models varies from 0.02 to 0.40 mm and from 0.12 to 0.40 mm, respectively. The systematic RMS varies significantly with the different OTL models, which shows that the systematic of the N2 and Q1 tidal constituents was mainly caused by the uncertainties of the OTL models. The uncertainties of the OTL models result in the systematic differences. After removing the systematic differences, the RMS of the residual phasors of the N2 and Q1 tidal constituents are all around 0.2 mm. The residual RMS does not change with the different OTL models, so the residual RMS reflects the uncertainty of the GPS estimates.

As shown in Figure 5, the systematic RMS change of the M2 and O1 tidal constituents with the various OTL models is from 0.01 to 1.02 mm and 0.20 to 1.01 mm, respectively. These results indicate that the systematic RMS of the M2 and O1 constituents was mainly caused by the uncertainties of the OTL models. After removing the systematic differences, the residual RMS of the O1 tidal constituent was around 0.2 mm, and no longer changed with the various OTL models. The residual RMS values of the M2 tidal constituent between the GPS estimates and the six OTL models of GOT4.10c + STW, FES2014b + STW, FES2014b + PREM, TPXO.8 + PREM, NAO99b + GB, and OSU12 + GB were all 0.2 mm. However, the residual RMS of the M2 tidal constituent between the GPS estimates and the other three OTL models (DTU10 + GB, EOT11a + GB, HAMTIDE + GB) were around 0.35 mm. This indicates that the residual RMS of the M2 tidal constituent changes slightly with the different OTL models. Therefore, the residual RMS should absorb the errors of the three OTL models, which may not exist entirely as systematic differences, because the M2 tidal resolution of the DTU10, EOT11a, and HAMTIDE ocean tide models is 1/8° × 1/8° smaller than the 1/2° × 1/2° size of Hong Kong. Overall the uncertainty of the GPS-estimated M2 tidal constituent is at the 0.2 mm level. 

Figure 5 also shows that there was obvious systematic RMS of the K1, P1, and K2 tidal constituents between the GPS estimates and the nine OTL models, of around 6 mm, 2 mm, and 9 mm, respectively. This indicates that the K1, K2, and P1 tidal constituents are affected by the significant common-mode errors for all the stations. These errors may include: (1) the unmodeled body tide; (2) the OTL model uncertainty caused by the ocean tide model and mantle inelasticity; and (3) GPS-related error, such as satellite orbit error, which has almost the same effect for all stations. Firstly, for the three tidal constituents of K1, K2, and P1, the unmodeled body tide error should be at the sub-millimeter level [12]. Secondly, the OTL displacement differences for the K1, P1, and K2 tidal constituents among the predictions by the nine different OTL models are at the sub-millimeter level, which indicates that that the OTL models are unlikely to have caused such misfits. Therefore, the common-mode errors are likely caused by the GPS-related errors. After removing the systematic differences of the K1, P1, and K2 tidal constituents, the residual RMS was around 0.8 mm, 0.5 mm, and 1 mm, respectively. The residual RMS was still significant, which indicates that these three tidal constituents are probably affected by the station-related error. Previous studies [13,14] inferred that K1 and K2 may be affected by the satellite orbit error and multipath effect, because the period of the K1 coincides with that of the satellite constellation, and the period of the K2 is almost the same as the satellite orbital period, while P1 may be disturbed by residual GPS signal propagation errors or non-tidal daily cycles in the atmosphere because its period is very close to solar day.

**Figure 6:**
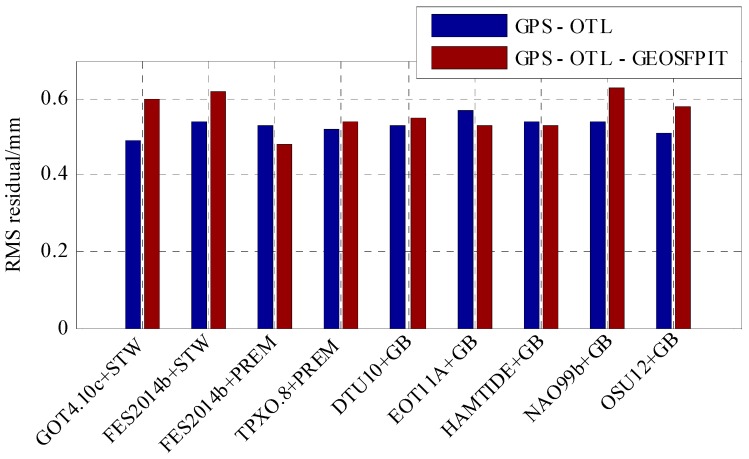
RMS residual of the S2 tidal constituent between the GPS estimates and the OTL models

As shown in Figure 5, the total RMS of the S2 tidal constituent between the GPS estimates and the nine OTL models were between 0.5 to 0.6 mm. There are some not obvious systematic differences of 0.1 to 0.3 mm between the GPS estimates and the nine different OTL models, which may arise from the effect of the atmospheric S2 tide. The effect of the atmospheric S2 tide was analyzed using the GEOSFPIT atmospheric model provided by the International Mass Loading Service [8], with a resolution of 0.625° × 0.5°. The results show that the amplitude of the atmospheric tide displacement, as predicted by the GEOSFPIT model, is only 0.22 mm at Hong Kong. Figure 6 shows that, after correcting the atmospheric tide by the GEOSFPIT model, the RMS residual of the S2 tidal constituent between the GPS estimates and some OTL models slightly reduced. Overall there was still an obvious RMS residual of about 0.5 mm between the GPS estimates and the OTL models. Figure 5 shows that the residual RMS of the S2 tidal constituent were all nearly 0.5 mm. This indicates that there are some station-related errors of around 0.5 mm, which arise from GPS-related error. GPS signal propagation error or non-tidal daily cycles in the atmosphere may bias the estimate of S2 because the period of S2 is half of the solar day [13,14]. This leads to the uncertainty of GPS-estimated S2 tidal constituent being at 0.5 mm level.

## 4. Conclusions

In this study, we used GPS kinematic PPP to estimate the vertical OTL displacements of eight semidiurnal and diurnal tidal constituents with 10-year GPS data from 12 GPS reference stations in Hong Kong. The systematic and residual phasor differences between the GPS estimates and nine OTL models were obtained by least squares methods in the complex domain. Our experimental results suggest that:

For the M2, N2, O1, and Q1 tidal constituents, the RMS residual between the GPS KPPP estimates and the different OTL models is mainly due to the uncertainties of the OTL models, which exist as a systematic component. When the systematic differences are removed, the residual RMS is basically within 0.2 mm, which indicates that the uncertainty of the four KPPP-estimated tidal constituents is at the 0.2 mm level. The K1, P1, and K2 tidal constituents are affected by significant GPS-related common-mode error. After removing the systematic differences, the residual RMS was still significant, which indicates that the three tidal constituents are also affected by GPS-related residual error. The RMS residual of the S2 tidal constituent between the GPS estimates and the OTL models is mainly caused by GPS-related error. Overall the uncertainty of the GPS-estimated S2 tidal constituent is at the 0.5 mm level. These results indicate that GPS KPPP can provide constraints for the Earth’s vertical tidal displacement at the 0.2 mm level for the M2, N2, O1, and Q1 tidal components, and the 0.5 mm level for the S2 tidal component. 

## Figures and Tables

**Table 1:** The periods of the eight semidiurnal and diurnal tidal constituents.

Name	Harmonic Constituent	Period/h
M2	Principal lunar semi-diurnal	12.4206
S2	Principal solar semi-diurnal	12.0000
N2	Larger lunar elliptic semi-diurnal	12.6583
K2	Luni-solar declinational semi-diurnal	11.9672
K1	Luni-solar declinational diurnal	23.9345
O1	Principal lunar declinational diurnal	25.8193
P1	Principal solar declinational diurnal	24.0659
Q1	Lunar elliptic diurnal	26.8680

**Table 2:** WGS84 coordinates and the Gross Error elimination rate for the 12 stations.

Station	Longitude (E)	Latitude (N)	Ellipsoidal Height/m	Data Length/d	% of Gross Errors
HKFN	114°08′17″	22°29′41″	41.212	3628	1.57%
HKKT	114°04′00″	22°26′41″	34.576	3640	1.06%
HKLT	113°59′48″	22°25′05″	125.922	3650	1.23%
HKMW	114°00′11″	22°15′21″	194.946	3529	4.58%
HKNP	113°53′38″	22°14′57″	350.672	3643	1.15%
HKOH	114°13′43″	22°14′52″	166.401	3646	1.08%
HKPC	114°02′16″	22°17′05″	18.130	3638	1.23%
HKSC	114°08′28″	22°19′20″	20.239	3594	2.61%
HKSL	113°55′41″	22°22′19″	95.297	3652	0.92%
HKSS	114°16′09″	22°25′52″	38.713	3645	1.11%
HKST	114°11′03″	22°23′43″	258.704	3651	0.93%
HKWS	114°20′07″	22°26′03″	63.791	3652	0.89%

## Appendix A

The amplitudes and phases of vertical ocean tide loading (OTL) displacements for eight semidiurnal and diurnal tidal constituents determined by GPS kinematic precise point positioning (KPPP) were shown in Table A1 and Table A2, in which all phases are positive for the Greenwich lag.

**Table A1:** KPPP-estimated vertical OTL displacements of four semidiurnal tides.

	M2		S2		N2		K2	
Station	A (mm)	Φ (°)	A (mm)	Φ (°)	A (mm)	Φ (°)	A (mm)	Φ (°)
HKFN	4.86	189.2	1.47	181.4	1.04	169.4	8.14	14.7
HKKT	5.25	187.8	2.03	200.0	1.10	175.0	7.32	30.6
HKLT	5.41	189.7	1.70	193.2	1.39	181.1	7.79	23.1
HKMW	5.88	192.9	1.41	193.0	1.44	182.7	8.04	14.7
HKNP	6.13	194.5	2.34	195.6	1.27	192.7	8.31	21.3
HKOH	6.17	189.8	2.09	207.3	1.50	181.1	7.80	31.9
HKPC	5.73	191.1	1.56	210.2	1.54	184.9	7.95	15.2
HKSC	5.82	189.9	1.94	189.7	1.42	182.5	11.55	18.4
HKSL	5.80	193.6	2.08	230.1	1.40	184.6	9.00	17.5
HKSS	5.55	184.9	1.79	190.7	1.33	179.9	7.69	31.7
HKST	5.44	184.8	2.02	178.3	1.34	176.9	8.09	20.1
HKWS	5.51	185.5	2.08	186.7	1.22	179.9	8.90	31.5

**Table A2:** KPPP-estimated vertical OTL displacements of four diurnal tides.

Station	K1		O1		P1		Q1	
	A (mm)	Φ (°)	A (mm)	Φ (°)	A (mm)	Φ (°)	A (mm)	Φ (°)
HKFN	7.21	32.7	6.73	306.9	1.85	260.2	1.20	297.4
HKKT	7.95	35.8	6.96	305.5	1.98	262.4	1.64	281.6
HKLT	6.47	40.6	7.25	304.4	1.00	255.8	1.62	283.9
HKMW	6.85	39.6	7.77	306.6	1.22	297.5	1.93	284.1
HKNP	8.21	32.5	7.76	308.8	1.17	260.8	1.73	291.4
HKOH	8.84	32.9	7.86	306.9	1.71	296.5	1.66	282.2
HKPC	8.03	32.1	7.46	306.0	1.38	300.2	1.79	281.4
HKSC	8.20	37.7	7.50	309.3	1.07	311.2	1.70	284.2
HKSL	8.08	41.7	7.63	308.1	1.74	289.5	1.53	286.3
HKSS	7.46	27.6	7.07	306.7	0.96	254.1	1.47	284.1
HKST	7.22	26.8	7.27	307.6	1.60	291.3	1.37	280.9
HKWS	7.20	32.5	7.30	305.3	1.50	268.3	1.35	276.5
